# Data integrity systems for organ contours in radiation therapy planning

**DOI:** 10.1002/acm2.12353

**Published:** 2018-06-12

**Authors:** Veeraj P. Shah, Pranav Lakshminarayanan, Joseph Moore, Phuoc T. Tran, Harry Quon, Curtiland Deville, Todd R. McNutt

**Affiliations:** ^1^ Department of Radiation Oncology and Molecular Radiation Sciences Johns Hopkins University School of Medicine Baltimore MD USA; ^2^ Department of Biomedical Engineering Johns Hopkins University Baltimore MD USA; ^3^ Department of Medical Oncology and Urology Johns Hopkins University School of Medicine Baltimore MD USA

**Keywords:** clinical workflow, contiguous, data integrity system, normal tissue contour, radiotherapy

## Abstract

The purpose of this research is to develop effective data integrity models for contoured anatomy in a radiotherapy workflow for both real‐time and retrospective analysis. Within this study, two classes of contour integrity models were developed: data driven models and contiguousness models. The data driven models aim to highlight contours which deviate from a gross set of contours from similar disease sites and encompass the following regions of interest (ROI): bladder, femoral heads, spinal cord, and rectum. The contiguousness models, which individually analyze the geometry of contours to detect possible errors, are applied across many different ROI's and are divided into two metrics: Extent and Region Growing over volume. After analysis, we found that 70% of detected bladder contours were verified as suspicious. The spinal cord and rectum models verified that 73% and 80% of contours were suspicious respectively. The contiguousness models were the most accurate models and the Region Growing model was the most accurate submodel. 100% of the detected noncontiguous contours were verified as suspicious, but in the cases of spinal cord, femoral heads, bladder, and rectum, the Region Growing model detected additional two to five suspicious contours that the Extent model failed to detect. When conducting a blind review to detect false negatives, it was found that all the data driven models failed to detect all suspicious contours. The Region Growing contiguousness model produced zero false negatives in all regions of interest other than prostate. With regards to runtime, the contiguousness via extent model took an average of 0.2 s per contour. On the other hand, the region growing method had a longer runtime which was dependent on the number of voxels in the contour. Both contiguousness models have potential for real‐time use in clinical radiotherapy while the data driven models are better suited for retrospective use.

## 1 INTRODUCTION

The concept of data integrity systems for mapped organ contours in radiation therapy aims to improve both the accuracy and consistency of data. New advances in automated segmentation technology paired with radiotherapy dose calculations have improved the ability of clinicians to accurately contour boundaries of organs at risk in radiotherapy.^1^, ^2^, ^3^, ^4^, ^5^, ^6^ These technologies have been applied to numerous regions of interest (ROI) in the head, neck, thorax, and prostatic regions.^7^, ^8^, ^9^ Other studies have segmented CT images using patient and population based statistics.^10^ However, with any qualitative, manually completed activity, there are margins of error, which if not detected, can have implications on the treatment of patients and how physicians treat future patients. Poorly or spuriously mapped contours by physicians and residents has the potential to result in erroneous radiation dosing of critical, noncancerous anatomy and has the potential to skew predictive models developed by data scientists to extrapolate post‐treatment parameters, such as weight loss and dysphagia.

Currently, many studies have been completed regarding integrity checking in radiation therapy across a given set of patients.^11^, ^12^ After treating patients with a consistent diagnosis, physicians tend to assess the variation in treatment planning and delivery across the set of patients with the goal of standardizing treatment for that diagnosis. Similar studies analyze the integrity of radiation treatment through a standardized set of parameters. Other studies aim to improve the safety and integrity of treatment through the verification of prescriptions and a variety of “in house parameters.”^13^ With regard to automated treatment control, studies have analyzed the efficacy of tools to improve the safety and integrity of intensity modulated radiation therapy (IMRT) while finding the optimum treatment plans for patients.^14^

On the other hand, this technology employs an active approach to improving integrity, through the lens of a clinical database environment. In this study, we developed two classes of contour integrity checks. The first, a data driven integrity check, aims to develop and test models which identify contours which deviate from the norm of a set of data. The second is an internal ROI check which is developed independently and applied to a set of contours with potential for real time applications in radiotherapy. By employing metrics to detect poorly contoured anatomy within the radiation oncology clinical workflow, this technology distinguishes itself from prior studies and technologies within the realm of radiation therapy planning integrity. It aims to improve the quality of clinical data for data scientists and physicians, minimizing the risk of radiation overdose to critical anatomy for patients.

## 2 MATERIALS AND METHODS

The contour data used in this study comes from the Oncospace^14^ database, a learning health system comprised of clinical radiotherapy patient data. Specifically, given the regions of interest tested in this study, we used contour data from the Oncospace Head and Neck and Oncospace Prostate databases. The Oncospace data used within this study was collected across several clinics to ensure accuracy and consistency throughout development and analysis. The number of contours analyzed is dependent on the region of interest and was not consistent across each region of interest. The development and testing of algorithms as well as analysis was completed using Python and MATLAB R2017a. In this study, we divide our models into two classes: data driven and contiguousness. With regard to the data driven models, we used several metrics as thresholds and classifiers to create the models.

The first metric used in the data driven models was total ROI volume. Using Microsoft Visual Studio as a platform, we used SQL direct queries to query patients from the Oncospace^15^ Prostate Database based on specific Region of Interest Volume. We then consolidated the patient lists, organizing by ROI, and exported the data into MATLAB and Python, our analysis software. Total ROI volume aims to detect abnormally large or abnormally small organ contours. To the analyst, an abnormally large organ contour could indicate incorrectly contoured surface anatomy while an unusually small volume could indicate missing geometric contour slices.

**Figure 1:**
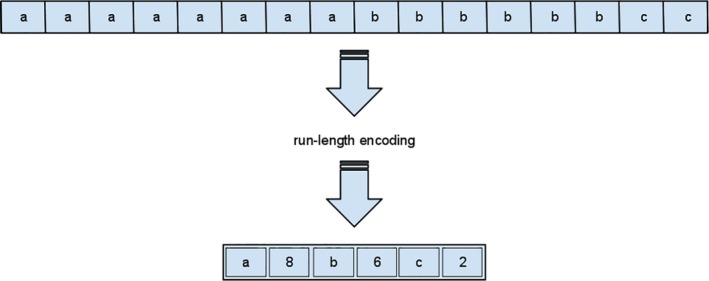
Shows a diagram explaining the concept of run length encoding. Run length encoding is a lossless data compression method which stores long runs of data into single data counts. A common application of this methodology is in JPEG files.

Similarly, the next metric, total ROI Extent, indicates anatomy which extends abnormally in the left‐right, anterior‐poster, or inferior‐superior directions. We define Extent as the range of voxels in a three‐dimensional grid in each direction. Total ROI Extent is calculated by converting the transition points of the binary mask of a contour into sets of Indices which map the surface voxels of this contour. The binary mask is encoded using a data compression technique called run length encoding,^16^ shown in Figure 1. Here, extensive runs of data are stored as single data counts rather than its original run. Then, we applied the following equations over each contour to compute indices for each contour. Below are the defined variables within the equations.

*X*dim: The voxel dimension factor in the left‐right direction.

*Y*dim: The voxel dimension factor in the anterior‐posterior direction.

*Z*dim: The voxel dimension factor in the inferior‐superior direction.

Mask: An array of run length encoded transition points of the binary mask of a contour.

Index: A certain transition point within the “Mask” array

(1) $$Z\;Indices=\frac{Mask\left(\mathrm{Index}\right)}{Xdim\times Ydim}$$

(2) $$Y\;Indices=\frac{Mask\left(\mathrm{Index}\right)-\left(\mathrm{ZIndices}\right)\times \left(\mathrm{Xdim}\right)\times \left(Ydim\right)}{\mathrm{Xdim}}$$

(3) $$Indices=Mask\left(\mathrm{Index}\right)-\left(\left(\mathrm{ZIndices}\right)\times \left(\mathrm{Xdim}\right)\times \left(\mathrm{Ydim}\right)\right)-\left(\left(\mathrm{YIndices}\right)\times \left(\mathrm{Xdim}\right)\right)$$

We then apply an algorithm to ensure that voxels fill each slice of the contour. Lastly, we subtract the maximum indices value from the minimum in each direction within a 3D space to find the Extent.

An abnormally large left‐right or anterior‐superior Extent could indicate suspicious or poorly contoured anatomy or missing slices in the inferior‐superior direction. While total Extent will detect more generalized abnormalities throughout the contour, sliced based Extent will detect specific abnormalities in Extent on a slice‐by‐slice basis throughout the mapped contour. For example, slice based Extent could detect frame shifts within a spinal cord or clearly missing slices. In addition, this metric could detect contractions in the anterior‐posterior direction, indicating suspicious contours or missing slices in the inferior‐superior direction. Another prominent application of slice based Extent is through the average slice based Extent and the range of slice based Extent in the left‐right and anterior‐superior directions over a region of interest.

The data driven models developed and tested in this study are over the following ROIs: bladder, femoral heads, rectum, and spinal cord. In these models, we combined the aforementioned metrics to develop thresholds which could be applied over sets of treated radiotherapy contours derived from CT imaging.^1^ These models distinguish themselves from the contiguousness models in that they are data driven, meaning they are tested and developed on a data set to optimize their ability to detect poorly or suspicious contoured anatomy. Each model is unique to a certain ROI. On the other hand, the contiguousness models were developed to be tested across many ROI to detect suspicious contours across the set.

### 2.A Bladder contour integrity model

In radiotherapy, prostate cancer patients often times are required to maintain full bladders for scans and treatments. This tool aims to predict whether contoured bladder anatomy for prostate cancer patients indicates a full or empty bladder. This predictive integrity model utilizes the variables of volume and Extent ratios to develop thresholds through which patient lists are sorted. Total ROI volume allows us to gauge the size of a bladder contour with respect to other contours within a set of patients. Total ROI extent was calculated by converting binary mask transition points into indices and finding the range among indices. Then, Extent ratios were calculated by dividing the Extents as follows: $\frac{\mathrm{lateral}}{anterior-posterior},\frac{\mathrm{lateral}}{inferior-superior},\frac{anterior-posterior}{inferior-superior}$. This model was created using a development group of 150 patients selected randomly using a MATLAB dataset structure function.

**Figure 2:**
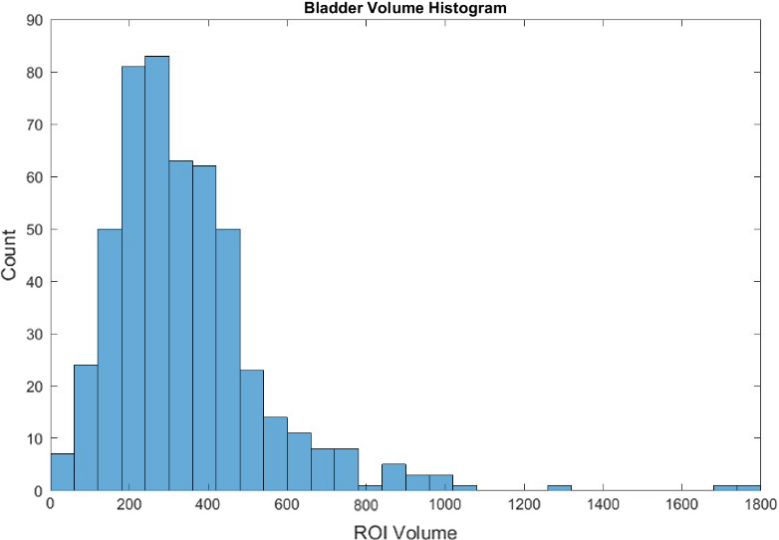
Shows a histogram of the volume of bladder contours. Once contour volumes were queried from the Oncospace database, the data were stored in an array and then projected into a histogram. This aims to show the shape and spread of bladder contour volume data.

**Figure 3:**
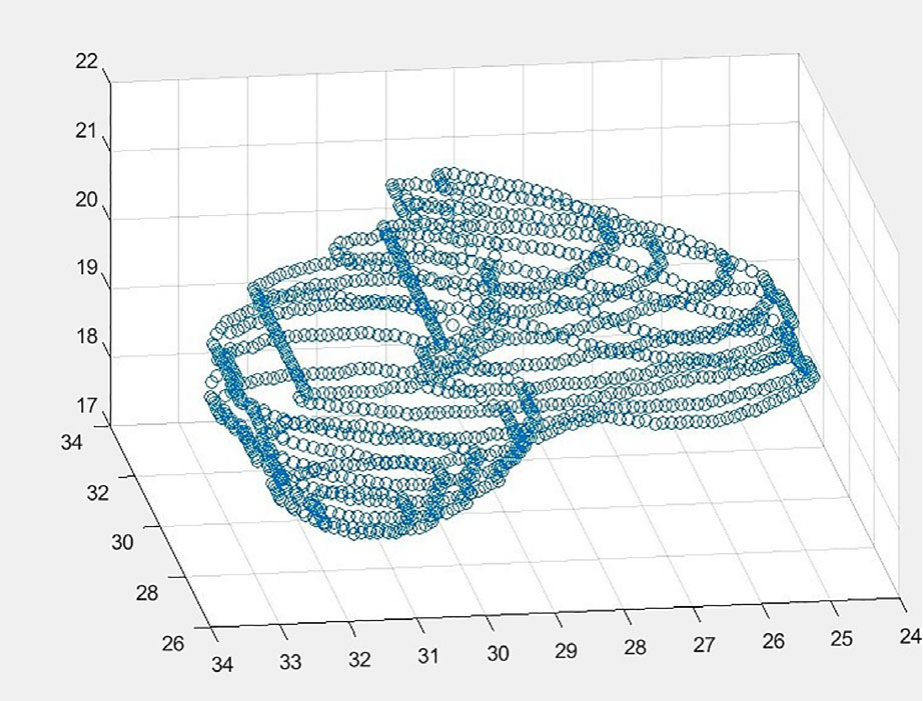
Shows a suspicious bladder contour detected by the bladder integrity model. Within this contour, one can see that it projects abnormally far in the anterior‐superior direction and seems to be missing contoured anatomy in the inferior‐superior plane.

The next set, 350 patients, was isolated as an experimental group. Using MATLAB for analysis, this model aims to isolate the bottom quartile of bladder volumes as an initial condition. Those which meet this condition are separated into a new array. Three arrays are created for contoured bladders which are below the 10th and above the 90th percentile for any of the three Extent ratios, as shown in Figure 2. The contours which met these conditions were then verified using an ROI shape verification tool which projects a surface plot of a contour, created using its indices derived from its mask and multiplied by a voxel dimension size. Figure 3 shows a suspicious bladder contour identified by the model.

### 2.B Femoral head integrity model

**Figure 4:**
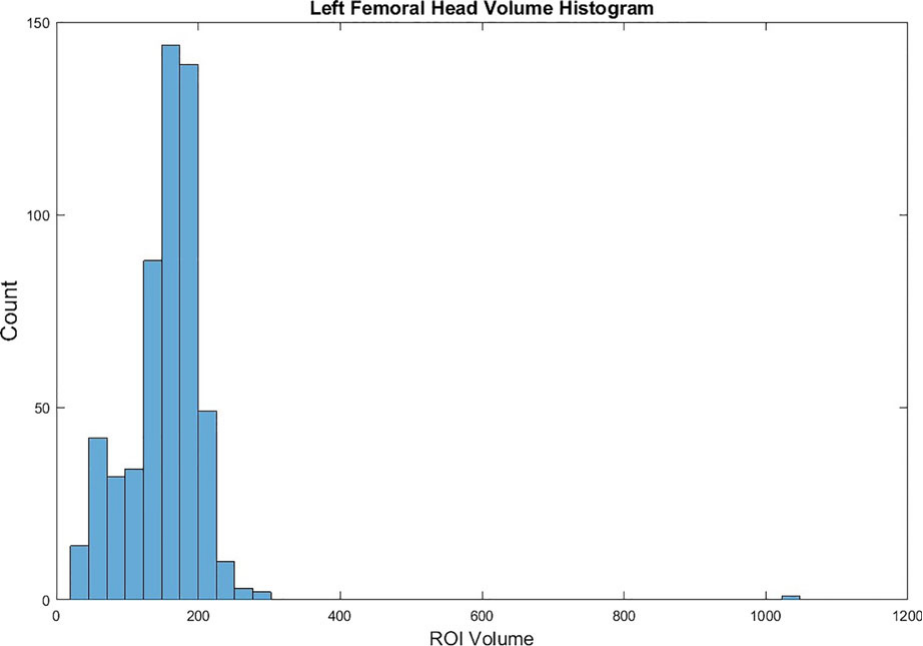
Shows a histogram of the volume of left femoral head contours. This histogram aims to show the shape and distribution of the volume of contoured femoral heads. Upon review, it seems to show a skew toward the lower end of volumes.

**Figure 5:**
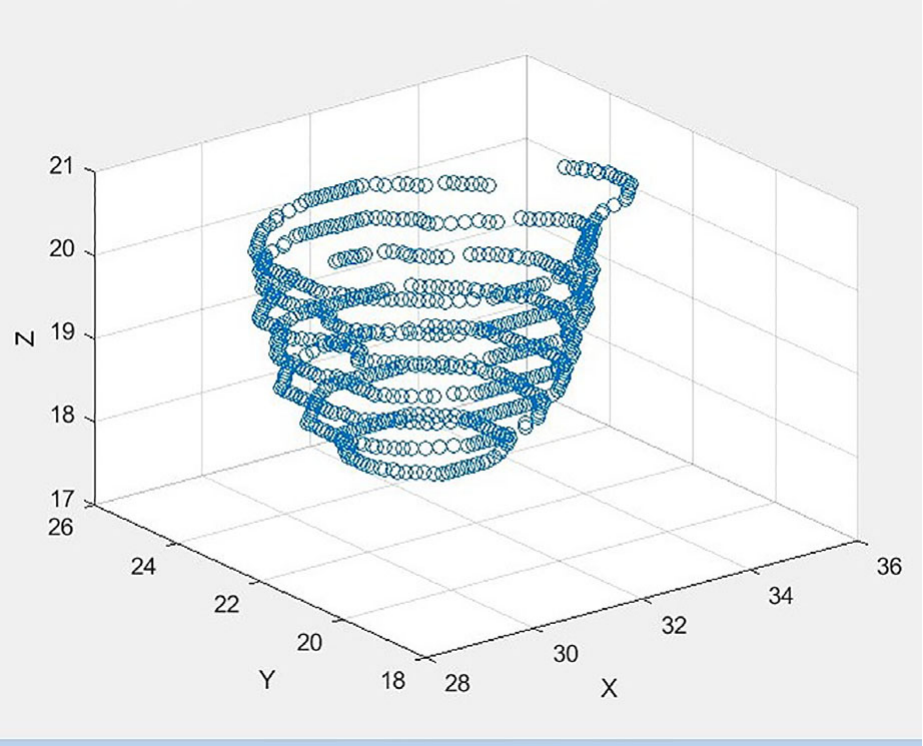
Shows a suspicious left femoral head contour using MATLAB software. One can see that only a small portion of the femoral neck is contoured and that the projected voxels in the inferior‐superior direction seem incomplete. This contour was clearly detected by the volume threshold set in this model.

When treating patients diagnosed with prostate cancer, femoral heads are often contoured in a clinical workflow as Organs at Risk of dose. The anatomy of femoral heads, composed of a head/neck and a shaft, often cause deviation in their contours. This model was developed to accurately distinguish between contours of the femoral head/neck and those of the femoral shaft. Using MATLAB for analysis, this model aims to detect contours which include the ball and shaft of the femoral head. Thus, those undetected by the model should only contour the ball of the femoral heads. Similar to the bladder emptiness prediction model, the femoral head integrity model was developed using a randomly selected development group and tested using a separate experimental group. The model utilizes an initial ROI volume threshold, isolating those volumes above the 20^th^ percentile, as shown in Figure 4. For the contours that met this condition, an iterative loop function with thresholds for $\frac{\mathrm{lateral}}{anterior-posterior}$ Extent ratio and inferior‐superior Extent was applied. A subarray of contours which met this condition were then verified using the same ROI shape verification tool used in the bladder contour integrity model to verify their suspicious nature. Figure 5 shows an example of a contoured femoral head, but not a femoral shaft.

### 2.C Rectum and spinal cord contour integrity model

Among ROI contours which possess variation from each slice in the inferior‐superior direction, slice based Extent calculations were used. The unique ability of slice based Extent is that it will detect poorly contoured anatomy which is not missing slices in any given direction but possesses abnormally oblong or undersized on any slice in the inferior‐superior plane. This is the case in rectum contours.

Using the Python development environment and MATLAB for development and analysis, we first isolated a development and testing group for each ROI. This integrity model iterates through slices of a radiotherapy contour along the inferior‐superior axis, calculating the Extent in both the left‐right and anterior‐superior directions, and saving this data into an array. It then calculates the range of Extents in the left‐right and anterior‐superior directions for each contour. Lastly, it creates another array by applying a function which sorts radiotherapy contours whose slice based Extents are greater than the 90th percentile in the left‐right and anterior‐superior directions. The contours within this array were then verified as abnormal using the ROI shape verification algorithm used in the bladder model.

### 2.D Contiguousness integrity model

This model, unlike the aforementioned models, is not data driven. Data were queried from the Oncospace Clinical Database and then run through the Contiguousness Integrity Model software. The sample size used for each Region of Interest varied based on the available contours in the Oncospace database. Contiguous contours possess surface voxels throughout their geometry and have geometry present across each slice throughout their bodies. The Contiguousness model is divided into two sub models: Contiguousness by Extent and Contiguousness by Region Growing over Volume.

The software supporting the contiguousness by Extent metric was derived from the concept that slice based Extent or total ROI volume would not be able to detect subtler suspicious contours. This model iteratively loops by slice across a region of anatomy and evaluates whether slices are missing in a given direction. Here, a contiguous contour is one in which number of unique voxels in each geometric direction plus one equals the Extent in that given direction. Noncontiguous contours would therefore be missing contoured slices in a given direction or possess voxels of contoured anatomy that is projected away from the main body of the contour.

**Figure 6:**
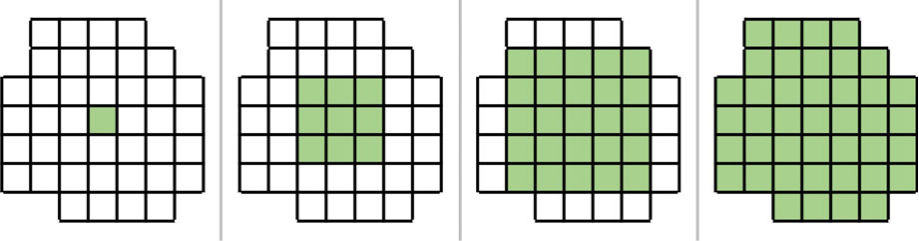
Shows a diagram which explains the characteristics of a contiguous contour. The diagram begins with one voxel filled and continues to grow until no more voxels can be filled without breaks, implying contiguity.

**Figure 7:**
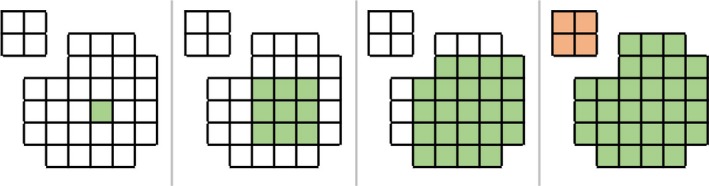
Shows a diagram which explains the characteristics of a noncontiguous contour. Here, one voxel fills the space and continues to grow. However, the number of voxels visited does not equal the number of voxels within the image.

**Figure 8:**
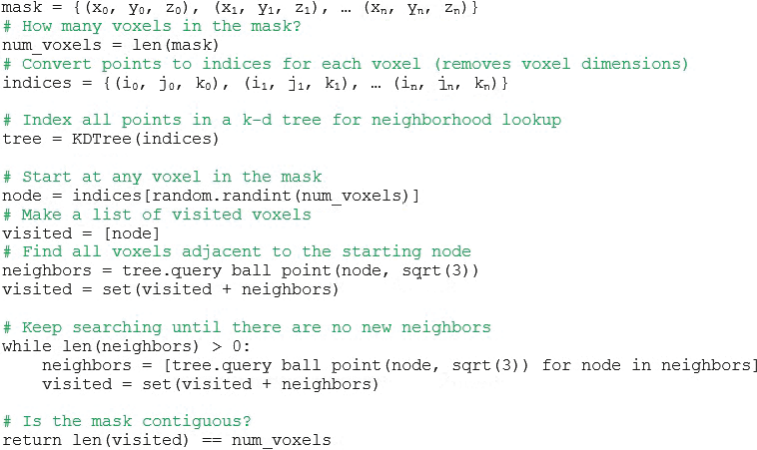
Shows a diagram of pseudocode explaining the region growing algorithm. Using the ball point query method, this code selects a voxel within the contour and continues to grow, finding all voxels within a given radius of the original. It does this throughout the entire contour as a test of contiguity.

The next contiguity metric, Region Growing over Volume, validates a binary mask structure over a voxel grid. Within this metric, contiguity is defined as having a path from one voxel to every other voxel throughout the mask of the contour. The name “region growing” is derived from the algorithm supporting contiguity via region growing over volume, which analyzes specific voxels within a binary mask. This algorithm was implemented in Python, using the SciPy library. Starting at a single voxel within a contour, this method repeatedly searches for all adjacent voxels until none exist. Figure 6 shows an example of a contiguous region, where the green voxel grows a neighborhood that encompasses all voxels in the structure. By contrast, in Figure 7, a noncontiguous region the number of visited voxels does not equals the voxels in image, indicating that there is not a contiguous path between all voxels of the structure. K‐d trees^17^ were used as a method for fast indexing and lookup of neighbors. The KDTree.query_ball_point()^18^, ^19^ method returns all points within a specified radius from a point, in this case, √3, since points are treated as a unit‐voxel grid corresponding to the voxel indices. Figure 8 shows pseudocode of the implementation of the region growing algorithm.

After creating these metrics, we applied their respective algorithm to patient lists across the aforementioned regions of interest. For the region growing algorithms, we also computed runtime data for the contours tested to assess their feasibility real time applications. Lastly, to check for false negatives, suspicious contours undetected by the integrity metrics, we conducted a blind review of all of the contours tested across the regions of interest. During this review, we marked all suspicious contours and then compared the results to that of the preliminary analysis.

## 3 RESULTS AND DISCUSSION

After completing the analysis, all data integrity models were successful at detecting suspicious or abnormal contours within the clinical workflow, however, to differing levels. Based on the results summarized in Tables 1, 2, 3, 4, 5, 6, it is clear that the Contiguousness models are the most exact method of detecting these contours within a clinical radiotherapy workflow as each suspicious contour was verified as abnormal via the MATLAB ROI shape verification tool. On the other hand, the data driven models, while accurate, did have false positives and false negatives. The results shown in Tables 1, 2, 3, 4, 5, 6 indicate that certain contours are contoured abnormally at a higher frequency than other contours. More so, Table 4 summarizes the accuracy of the data driven models, while Tables 5 and 6 summarizes the accuracy of the contiguousness models. A common example is the spinal cord, which was contoured suspiciously 82 times using a sample of 1148 patients. In the case of the spinal cord, a treated noncontiguous contour could result in radiation dose passing through the cord to critical, nontarget anatomy. This issue also presents in ROIs, such as the bladder, where the contoured anatomy is near the target volume.

**Table 1:** Results of data driven integrity models

Region of interest	Integrity model or metric	Contours tested	Contours detected as suspicious	Contours verified as suspicious	False negative suspicious contours
Bladder	CT emptiness verification	594	36	25	5
Right femoral head	Femoral head distinction model	559	235 Detected with Ball/Shaft; 115 Detected with Ball only	235 Ball/Shaft Verified; 94 Ball only verified	N/A
Left femoral head	Femoral head distinction model	561	274 Detected with Ball/Shaft; 76 Detected with Ball only	274 Ball/Shaft Verified; 65 Ball only verified	N/A
Rectum	Slice based extent model	1148	12	10	9
Spinal cord	Slice based extent model	1148	30	22	60

**Table 2:** Contiguousness by extent results

Region of interest	Contours tested	Contours detected as suspicious	Contours verified as suspicious	False negative suspicious contours
Bladder	594	8	8	2
Right femoral head	559	7	7	2
Left femoral head	561	13	13	2
Prostate	322	1	1	4
Rectum	769	14	14	5
Spinal cord	1148	80	80	2
Brainstem	1140	14	14	0

**Table 3:** Contiguousness by region growing over volume results

Region of interest	Number of contours tested	Contours detected as suspicious	Contours verified as suspicious	False negative suspicious contours
Bladder	594	10	10	0
Right femoral head	559	9	9	0
Left femoral head	561	15	15	0
Prostate	322	1	1	4
Rectum	769	19	19	0
Spinal cord	1148	82	82	0
Brainstem	1140	14	14	0

**Table 4:** Accuracy of data driven metrics

Region of interest	Metric	Accuracy of detected contours	False positive rate	False negative rate
Bladder	CT emptiness verification	70%	30%	16.7%
Right femoral head	Distinction model	100%; 82%	N/A	N/A
Left femoral head	Distinction model	100%; 86%	N/A	N/A
Spinal cord	Slice based extent model	73%	26.6%	73.1%
Rectum	Slice based extent model	83%	17%	47%

**Table 5:** Accuracy of contiguousness by extent

Region of interest	Accuracy of detected contours	False negative rate
Bladder	100%	20%
Right femoral head	100%	22%
Left femoral head	100%	13.3%
Spinal cord	100%	2.4%
Rectum	100%	26.3%
Prostate	100%	80%
Brainstem	100%	0%

**Table 6:** Accuracy of contiguousness by region growing over volume

Region of interest	Accuracy of detected contours	False negative rate
Bladder	100%	0%
Right femoral head	100%	0%
Left femoral head	100%	0%
Spinal cord	100%	0%
Rectum	100%	0%
Prostate	100%	80%
Brainstem	100%	0%

Using the Bladder Contour Data integrity model indicates a high level of variation in contoured bladder anatomy, giving way to nine false positive contours. For example, a bladder contour which is full but extends highly in the inferior‐superior axis could meet the thresholds of the Bladder Contour Integrity model, but upon review, it is clear that the bladder is actually full. Other contours, upon review, were simply abnormally small in volume yet clearly the bladder was full based on its spherical geometry. On the other hand, the bladder model possessed rather few false negatives compared to the other models. This is because the vast majority of empty bladders will either be detected by the volume of extent ratio thresholds. The majority of the detected bladders were clearly contoured as empty based on the verified geometry of the structure. This poses a significant consistency issue in a radiation oncology clinical workflow during CT.

The accuracy of the femoral head prediction model can be attributed to nature of femoral head radiotherapy contours. Due to the binary nature of these contours, using a model which categorizes contours based on volume, inferior‐superior Extent, and Extent ratios will clearly be successful. However, the contours poorly classified by the model can be attributed to the fact that a smaller portion of the femoral head could be contoured with the shaft, therefore failing to meet the volume threshold as shown in Table 4. False negative rates were not calculated as the point of this model was to distinguish between two types of femoral head contours. Both the femoral head and bladder models could be implemented as preliminary integrity checks within radiotherapy planning to warn clinicians before they treat a suspiciously contoured plan.

With regard to the Slice Based Extent Rectum and Spinal Cord Models, both models were successful in detecting abnormal slice Extents throughout a contour, having 73% and 83% accuracy respectively. Within the Spinal Cord model, we found less than 10% overlap in the contours detected by the contiguousness models and the Sliced Based Extent model, proving that they both detect unique cases of suspicious or abnormal contours. The false positives noted in both models can be due to extreme deviation in slice Extent of normal anatomy. For example, a patient with an abnormally arched spinal cord or one diagnosed with scoliosis would fall in the Extent threshold defined in the model. Another example would be a rectal contour which extends significantly in multiple directions, making a high range in slice based Extent probable. More so, as shown in Table 4, the 73.1% of false negatives present with the spinal cord model can be attributed to the fact that single missing slices in the contours will not significantly affect the values of slice based extent. Therefore, the slice based extent model will only see more significant discontiguities, such as those with multiple missing slices. Both of these models are important as suspiciously contoured slices with abnormal left‐right or anterior‐superior Extent possesses the same clinical risks as those highlighted in the bladder model.

**Figure 9:**
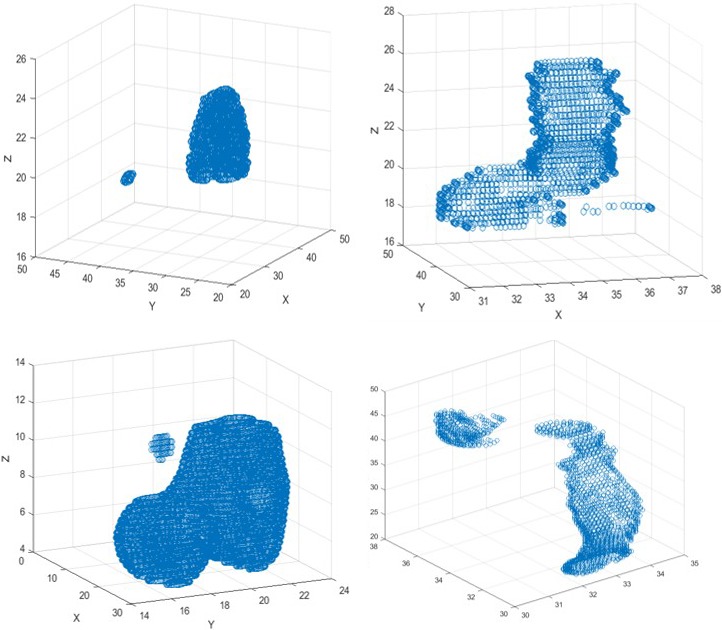
Shows a collection of suspicious contours detected by the contiguousness metrics from across datasets within the Oncospace database using MATLAB software. The contours include a bladder contour, a femoral head contour, a spinal cord contour, and a rectum contour.

Lastly, with regards to the nondata driven contiguousness metric, while both methods were quite accurate, it can be seen that each method poses its own benefits and drawbacks. As shown in Figure 9, both models detected suspicious contours. While the contiguousness by Extent model was the fastest, there were instances where it failed to detect suspicious contours which were detected using the region growing method. This is shown in Table 5 as this model yielded false negatives rates between 2.4% and 30% for the majority of regions of interest tested. The contiguousness by Extent model detects the most obvious suspicious contours, but sometimes fails to detect more nuanced abnormalities, which the region growing method detects. For example, it only successfully detected 14 of the 19 actually noncontiguous Rectum contours and 13 of the actually 15 noncontiguous left femoral head contours. The region growing method provides an improved robustness compared to the Extent‐measuring method, detecting between 2 and 5 additional suspicious bladder, rectum, femoral head, and spinal cord contours. The Extents method relies on projections along each dimension, checking that the structure is complete only along that dimension. However, it is possible that each projection may be contiguous even if the structure itself is not. The region growing method yielded false negatives only in one region of interest, prostate. This is because very oblong or exceedingly angled contours in the prostate region will not be detected by the region growing algorithm. This also explains why there is such a high false negative rate for prostate using the Extents method. With regards to runtime, the extents method had an average of 0.2 s, making it very feasible for real time applications in clinical radiotherapy.

**Figure 10:**
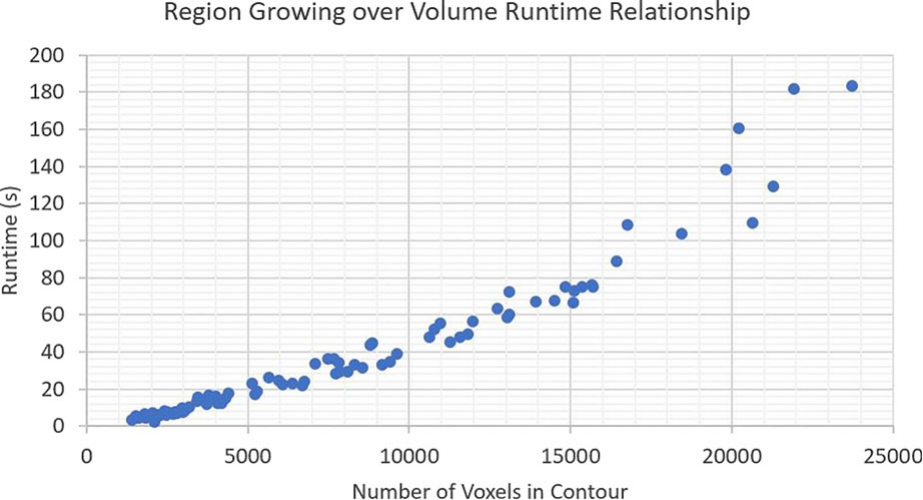
Shows a plot describing the relationship between the runtime of tested contours and number of voxels in the contour for the Region Growing algorithm. This plot shows a direct relationship between the two variables.

Additionally, while the region‐growing model is highly accurate, the implementation often results in a high runtime due to the exponential computational complexity of the algorithm, taking between 30 s and a minute to check a single ROI contour. However, an average runtime cannot be cited for this method as the runtime is wholly dependent on the numbers of voxels present in the contour, shown in Figure 10. As shown in Table 6, the Region growing model successfully verified each suspicious contour due to the robust and precise nature of its algorithmic development. On the other hand, the compared simplicity of the contiguousness by Extent model would allow it to have implications for use in real time in a radiotherapy clinical workflow. While contouring patient anatomy for treatment, physicians could utilize this model to ensure integrity of contours in real time, thus potentially increasing the efficacy and safety of treatment.

Another important strength of the region growing method is associated with the smoothness assumption. In general, anatomical structures are smooth, meaning that between slices in a 3D representation, voxels in neighboring slices should be close together. In the occurrence of an irregular structure or contour, where the rings defining the edge of each axial slice are too far apart, the region growing over volume will not deliver a false positive as it is able to travel between slices along the overlapping interior voxels.

Overall, the suspicious contours detected by the aforementioned methods are present for several reasons. The main reason is due to human error in the contour process. The contours used in analysis were collected across several physicians and clinics to ensure that the suspicious contours could not be attributed to a single physician. There are no clear biases in contour data that would allow certain contours to better fit certain integrity models.

## 4 CONCLUSION

The models developed and tested in this study each have benefits. The data driven models are effective in finding specific cases of contours but, due to their lesser accuracy and more significant false negative percentages, are more suited for retrospective analytics. On the other hand, the contiguousness models are both suited for real time clinical use due to their zero false positive and minimal false negative percentages. While the region growing algorithm does have a significantly longer runtime when compared to the extents model, it still is feasible for real time use and could be improved for a faster runtime in future iterations.

More so, this study shows the need for contour integrity system in clinical radiotherapy during the planning process. Potentially, such a tool could be used in conjunction with CT and Atlas based auto‐segmentation methodologies. This will not only minimize the risk of radiation overdose to critical anatomy in a clinical workflow but aid physicists, clinicians, and data scientists in the creation of post‐treatment predictive models with the goal of further improving patient care.

## CONFLICT OF INTEREST

There are no conflicts of interest.
